# Implementing Artificial Intelligence in Daily Clinical Practice, from Voice Dictation to Operating Room Organization: A Targeted Narrative Review with Hysteroscopy as a Clinical Exemplar

**DOI:** 10.3390/jcm15218688

**Published:** 2026-11-07

**Authors:** Sergio Haimovich, Afchine Fazel

**Affiliations:** 1 Department of Obstetrics, Gynecology and Reproduction, Dexeus University Hospital, Gran Via Carles III 71–75, 08028 Barcelona, Spain; 2 Clinique Bizet, 23 Rue Georges Bizet, 75116 Paris, France; a.fazel@aphp.fr; 3 Department of Obstetrics and Gynecology, Hôpital Lariboisière, 2 Rue Ambroise Paré, 75010 Paris, France

**Keywords:** artificial intelligence, ambient clinical documentation, clinical decision support, hysteroscopy, validation, operating room management, implementation science, patient safety, narrative review

## Abstract

Artificial intelligence (AI) is entering routine care through documentation tools, decision support, and hospital operations. The central question is whether these systems improve clinical work and patient care once embedded in routine workflows, not only whether they perform well on test data. This targeted narrative review follows AI from voice dictation and ambient documentation to operating room (OR) organization, using hysteroscopy as a clinical exemplar of the transition from technical performance to clinical utility. Evidence identified through targeted searches completed on 6 September 2026 was appraised with a framework that distinguishes image-level from patient-level results, internal from independent validation, and discrimination from clinical utility. Randomized trials and multisite observational data show modest, product-dependent benefits of clinician-reviewed ambient documentation; inaccuracies persist. Evidence for patient-message drafting derives from ratings of responses to public-forum questions and supports only bounded, supervised feasibility testing. In hysteroscopy, selected models discriminate well in narrowly defined tasks, but most studies are retrospective, image-level, or internally validated, and patient-level errors in high-risk categories are seldom reported. Reported gains in OR scheduling come largely from simulation. Safe implementation requires a defined clinical need, baseline measurement, subgroup-aware local validation, accountable review, and predefined criteria for expansion or withdrawal.

## 1. Introduction

Clinicians need tools that return time to patient care and reduce cognitive load. Documentation burden, fragmented electronic health records (EHRs), and inefficient use of operating room (OR) capacity make AI appealing, but performance in isolation says little about what happens when a system enters a real service. Its value depends on the clinical task, the intended users, the available information, and the consequences of error. Broad reviews of AI in health care describe substantial opportunities while emphasizing the need to evaluate effectiveness in the setting of use [1,2].

Several technologies are often grouped under the same label. Predictive machine learning estimates outcomes from patterns in structured or unstructured data, such as procedure duration or cancellation risk. Computer vision identifies or localizes features in images and video. Generative AI, including large language models (LLMs), produces text such as draft notes, summaries, and patient messages. Ambient documentation combines speech recognition with language processing and, frequently, generative summarization. Prescriptive analytics uses predictions and explicit constraints to recommend actions, such as a proposed surgical schedule. These categories overlap, but their validation requirements differ.

This targeted narrative review follows AI along the daily clinical workflow, from voice dictation to OR organization. It is neither a systematic review nor a comprehensive map of clinical AI applications; it examines selected applications that illustrate the different kinds of evidence a clinical service needs before adoption. Hysteroscopy is used as the specialty exemplar for three reasons. First, it is a digital video procedure in which a single clinical episode generates text (indications, operative reports, and patient communication), images (diagnostic and operative views), and scheduling demands, so it touches every workflow stage examined here. Second, published hysteroscopic AI spans diagnostic classification, preoperative planning, and prognosis, allowing different translational steps to be compared within one clinical domain. Third, a recent systematic review with formal risk-of-bias assessment provides a documented evidence base against which the primary studies can be examined [3]. Hysteroscopy is therefore used to illustrate how technical performance should be interpreted before clinical use, not to represent minimally invasive gynecologic surgery as a whole.

The review addresses three questions. (1) For AI applications across the daily workflow, from documentation to OR organization, what evidence exists of effects in routine use, as distinct from technical performance, simulated benefit, or proposed use? (2) Using hysteroscopy as an exemplar, to what extent do reported performance metrics support patient-level clinical conclusions, given the unit of analysis, the independence of validation, and the reporting of clinically relevant errors? (3) What governance, safety, and local evaluation requirements follow for services considering adoption? The scope is deliberately practical: the review is intended to support local evaluation, not to serve as a clinical practice guideline or product endorsement. Human oversight is necessary throughout, but supervision alone does not establish safety.

## 2. Methods

### 2.1. Design

We conducted a targeted narrative review structured around the three questions stated in Section 1. No systematic-review protocol was registered or required, and the searches were directed toward these questions rather than designed to retrieve every eligible publication.

### 2.2. Literature Identification and Selection

Literature identification and citation verification were completed on 6 September 2026, using PubMed-indexed records accessed through PubMed and Europe PMC, publisher websites, Crossref bibliographic records, and official regulatory and guidance sources. Searches combined terms related to AI with terms related to the clinical workflow, including “ambient AI scribe,” “clinical documentation,” “speech recognition,” “hysteroscopy,” “endometrial lesions,” “hysteroscopic myomectomy,” “intrauterine adhesions,” and “operating room scheduling.” Reference lists of relevant reviews were used to identify primary reports. The exact search strings, the numbers of records retrieved, and individual screening decisions for this targeted search were not recorded in a screening log; record counts therefore cannot be reported for the original selection.

Sources were selected when they (a) evaluated or reviewed an AI application relevant to one of the workflow stages examined (documentation, summarization, patient communication, operative reporting and coding, preoperative planning, hysteroscopic image analysis or prognosis, and OR management); (b) reported quantitative performance, workflow, or patient outcomes, or provided reporting, appraisal, regulatory, or governance guidance; and (c) were peer-reviewed publications or official documents. Marketing material was not treated as evidence of effectiveness. When several sources addressed the same question, we prioritized systematic reviews, randomized studies, multisite implementation studies, and primary diagnostic or prognostic studies, and, within a research line, the most complete report.

Study-specific numerical claims were checked against primary reports, using full text when accessible and indexed abstracts otherwise. Publisher and Crossref records were used to check citation details. Regulatory statements were checked against official sources, and the regulatory status of the European Union AI Act was rechecked against EUR-Lex.

### 2.3. Appraisal Approach

The hysteroscopy section is a secondary narrative synthesis informed by the systematic review of Watrowski et al. and supplemented by examination of the cited primary reports [3]. We did not independently rescore risk of bias, because a credible reassessment would have required duplicate assessment with the appropriate tools and complete access to every primary report, which was not available for all studies. Formal risk-of-bias judgments are therefore attributed to Watrowski et al., who used the Quality Assessment of Diagnostic Accuracy Studies 2 (QUADAS-2), the Prediction Model Risk of Bias Assessment Tool (PROBAST), and the revised Cochrane risk-of-bias tool for randomized trials (RoB 2).

Separately, we appraised each primary hysteroscopy study descriptively, using the common framework described in Section 4.1; this appraisal is not a formal risk-of-bias assessment. The full texts of all eight studies were examined. Items that could not be verified are labeled as such, and values taken from the secondary review are identified.

This review did not use duplicate screening, a registered protocol, a formal certainty-of-evidence grading system, or meta-analysis. Study design, unit of analysis, validation independence, comparator, and clinical relevance guided interpretation. The readiness categories presented in Section 7 are the authors’ qualitative judgments and should not be interpreted as validated evidence grades.

### 2.4. Use of Generative AI

Generative AI assisted editorial review, manuscript revision, reference identification and checking, extraction of study characteristics from primary reports, language editing, and review of the schematic figures, as disclosed in the Acknowledgments. Extracted data and generated suggestions were checked against the underlying publications and official sources. AI output was not used as evidence or to assign risk-of-bias judgments.

## 3. AI Applications Across the Clinical Workflow

### 3.1. Voice Dictation and Ambient Clinical Documentation

Voice dictation and ambient documentation address related but different tasks. Dictation converts deliberate clinician speech into text; an ambient scribe captures a conversation and generates a draft clinical note. Because an ambient scribe both transcribes and interprets the encounter, it can omit details, attribute information to the wrong person, or fabricate content. Evidence from older speech-recognition systems should therefore not be transferred directly to every contemporary LLM-based product.

In a longitudinal cohort study at five US academic health care institutions, Rotenstein et al. compared changes among 8581 clinicians, including 1809 who adopted AI scribes. Difference-in-differences estimates associated adoption with 13.4 fewer minutes of total EHR time (95% confidence interval [CI], 9.1–17.7) and 16.0 fewer minutes of documentation time (95% CI, 13.7–18.3), each normalized to eight scheduled patient hours. Visit volume increased by 0.49 visits per week (95% CI, 0.17–0.81); this weekly outcome did not use the time-based denominator. EHR time outside scheduled work did not change significantly. These findings are observational, and voluntary adoption at most sites leaves room for selection bias and residual confounding [4].

Randomized evidence supports a more differentiated interpretation than a general claim of improved productivity. Afshar et al. conducted a 24-week stepped-wedge trial involving 66 practitioners. Ambient AI reduced work exhaustion/interpersonal disengagement and time spent on notes, the latter by 0.36 h per day (95% CI, 0.17–0.55). Professional fulfillment did not meet the trial’s significance criterion, and the after-hours work result was sensitive to extreme observations [5]. In a three-group trial involving 238 physicians across 14 specialties, Lukac et al. compared two commercial ambient scribes, Microsoft Dragon Ambient eXperience (DAX v2.0) Copilot and Nabla (Nabla v1.5), with usual care. Nabla reduced time-in-note by 9.5% relative to control, whereas DAX did not produce a statistically significant reduction. Some secondary well-being measures improved, but clinically significant inaccuracies were reported occasionally [6]. These trials support supervised use in selected workflows while showing that benefits vary by product, adoption intensity, and outcome.

A prospective pilot involving 48 physicians reported improvements in perceived task load, burnout, and usability, although its design cannot establish causal or durable effects [7]. Systematic and scoping reviews identify variable transcription accuracy, errors in specialized terminology, and additional editing burden [8,9,10]. Word error rate is a transcription metric, not a direct measure of clinical harm; a single incorrect medication dose may matter more than several inconsequential word substitutions. Neither a low error rate nor favorable user satisfaction establishes patient safety.

For a clinical service, implementation starts with an approved platform, a clear explanation of the recording process, and a workable option for patients to decline. Requirements for consent and recording retention should be determined locally. Clinicians must verify medications, doses, allergies, negation, laterality, and the assessment and plan before signing. Evaluation should include total review and correction time, after-hours work, clinically consequential errors, patient experience, and clinician workload. Time saved should not automatically be converted into additional appointment capacity before its effect on workload and safety is understood.

### 3.2. Clinical Summarization and Chart Review

AI-generated summaries can make a fragmented record easier to review before a visit or handoff. Proposed uses include longitudinal problem summaries and assistance with medication reconciliation. Within the evidence examined here, outcome-level support for these uses is less developed than that for ambient documentation. A summary may be fluent yet omit an allergy, a previous treatment failure, or a relevant negative finding.

Maw et al. discuss implementation challenges in hospital medicine, including the use of general-purpose AI ahead of institutional support; their article is a commentary and should not be treated as a clinical effectiveness study [11]. Institutions should provide approved workflows that preserve access to the original record. Summaries should identify their source documents and dates, distinguish documented facts from generated interpretation, and permit verification of high-risk items. Patient-specific reconciliation remains a clinical task. Summary length or generation speed alone would not establish benefit.

### 3.3. Patient Communication, Education, and Discharge Instructions

LLMs can draft responses to portal messages, educational material, and discharge instructions. In a cross-sectional study of 195 questions posted to a public online forum, licensed health care evaluators preferred chatbot responses in 78.6% of evaluations. Ratings of good or very good quality were 3.6 times as prevalent, and ratings of empathetic or very empathetic responses 9.8 times as prevalent, for chatbot-generated responses as for physician responses [12]. These are relative frequencies of evaluator ratings, not measures of clinical effectiveness or patient satisfaction.

The study used public questions without a complete clinical history, professional rather than patient raters, and substantially longer chatbot responses. It did not test an implemented patient portal or measure health outcomes. Within the evidence examined here, therefore, drafting assistance has been evaluated only outside clinical workflows; its results justify a tightly bounded, supervised feasibility study in which every draft is reviewed and edited by a clinician before release, not routine use within a portal. Translation, reading level, urgency, treatment instructions, and warning symptoms require particular attention. Patient messages should have a clear escalation route; autonomous triage or unreviewed therapeutic advice is beyond the supervised drafting use discussed here.

### 3.4. Operative Reporting, Coding, Registries, and Audit

Language processing can structure operative notes, suggest codes, and extract variables for registries. These tools could reduce repetitive data entry, but their accuracy must be established for each task before the output is used elsewhere; otherwise, an error in the source note can be reproduced in billing, audit, and research datasets.

For hysteroscopic procedures, a useful reporting template may include the indication, relevant findings, procedure performed, tissue submitted, complications, and the distension-fluid record. An AI system should flag missing information rather than infer an undocumented finding or invent a fluid deficit. The surgeon should attest to the final report, and coding suggestions should undergo the usual coding validation process. Extraction accuracy should be audited against source records, with separate attention to missing values and false entries. These are proposed implementation safeguards rather than demonstrated benefits of a specific reporting product.

### 3.5. Operating Room Management and Organizational Efficiency

OR management involves linked constraints: procedure duration, staff availability, anesthesia resources, equipment, turnover, emergency demand, and postoperative capacity. Predictive models estimate individual events; optimization systems combine these predictions with constraints and priorities to propose a schedule. Better duration prediction does not automatically produce a better schedule or shorter patient waits. This section concerns organizational efficiency; other perioperative uses of AI, such as automated safety checklists and medication-administration monitoring, pursue different safety objectives and are outside its scope.

Systematic reviews describe applications to case-duration prediction, cancellation risk, and postanesthesia care unit demand [13,14]. Their findings support the feasibility of predictive assistance but do not establish that a model will retain its accuracy after transfer to a different hospital. Prediction errors should be compared with existing scheduling practices and simple statistical baselines, and performance should be examined by procedure, surgeon, case complexity, and urgency rather than only across the whole service.

Çalışkan et al. compared rule-based scheduling, predict-then-optimize methods, genetic algorithms, and reinforcement learning in a discrete-event simulation with 500 replications. Relative to the simulated baseline, reinforcement learning reduced total delay by 42.9%, overtime by 46.0%, and same-day cancellations by 54.8%, and utilization increased by 6.9 percentage points. The simulation represented a stylized general-surgery environment, not a prospective deployment at a particular institution, and its authors explicitly bounded the results by the modeled rewards, resource constraints, and disruption patterns [15]. These estimates describe simulated performance and should not be used as expected clinical savings in a business case. Within the evidence examined here, OR evidence therefore consists of systematic reviews of predictive models and one simulation study; no prospective deployment study was included.

Practical deployment must also accommodate events that disrupt a planned schedule. Emergency cases require protected capacity or explicit rules for displacing elective lists, and unexpected prolongation of a procedure, late cancellation, staff absence, limited anesthesia availability, or a shortage of postanesthesia care unit or ward beds can invalidate a recommendation generated earlier in the day. A scheduling system should therefore update its recommendations when these inputs change, represent clinical urgency explicitly, and show the decision-maker the consequences of a change, such as which cases would be delayed or canceled. Authority to reprioritize cases should remain with the designated clinical, anesthesia, and nursing coordinators, and overrides should be logged for evaluation. Within the evidence examined here, the handling of such disruptions has been studied only in simulation.

Local evaluation should first run in shadow mode, generating recommendations without changing actual care. Subsequent prospective testing should retain human scheduling authority and explicit clinical constraints, including urgency, equitable access, staff workload, and postoperative bed capacity. Institutions should monitor whether operational gains shift delays or burdens elsewhere in the care pathway. Autonomous scheduling that can materially alter access to treatment requires substantially stronger evaluation than a dashboard that suggests likely case duration.

Table 1 summarizes the principal workflow applications and the outcomes that local implementation should test. Table 2 summarizes selected quantitative evidence from the non-hysteroscopic domains and identifies whether each result was obtained in routine clinical practice, in a cross-sectional evaluation, or in simulation.

## 4. Evidence in Hysteroscopy

### 4.1. Evidence Base and Appraisal Framework

The systematic review by Watrowski et al. searched PubMed/MEDLINE and EBSCOhost databases to 8 March 2026, used duplicate screening, and included 19 primary studies spanning classification and detection, real-time localization, segmentation, operative guidance, and prognostic support [3]. Most were retrospective and single-center, external validation was limited, and only one randomized study assessed a procedural outcome. According to that review, nine studies were at high or high/unclear risk of bias, including all three prognostic studies assessed with PROBAST; eight were at moderate to moderate–high risk; and the single randomized trial had some concerns with RoB 2. Recurring problems were the absence of external validation, single-center retrospective design, and image- or frame-level rather than patient-level data splitting [3]. These are the review authors’ judgments, and they characterize the studies included in that review, not every gynecologic AI application.

Table 3 and Table 4 apply a common descriptive framework to the eight primary studies discussed below, based on three distinctions. Image-level metrics cannot be read as patient-level sensitivity unless patients are the unit of analysis. Random partitions of one database, even a multicenter database, constitute internal validation; independent external validation requires patients from centers, periods, or settings not used for model development. Discrimination, for example the area under the receiver operating characteristic curve (AUROC), does not establish clinical utility, which depends on error rates at a clinically chosen threshold, prevalence in the intended population, and the consequences of false-negative and false-positive results. Missing or unverifiable confidence intervals, thresholds, predictive values, and calibration are stated as such and were not derived from AUROCs.

### 4.2. Lesion Classification and Diagnostic Support

Wang et al. developed the ECCADx system to distinguish atypical endometrial hyperplasia (AEH) and endometrial cancer from benign findings, using 55,874 images from 1394 patients at three tertiary hospitals [16]. The model was trained on 1204 patients from one hospital (2008–2017) and tested on a later cohort from the same hospital (85 patients, 2018–2019) and on a cohort from two other hospitals (105 patients, 2019), with no case overlap reported. This design separates test data by patient, time, and, externally, center, although both test cohorts were retrospective. Reported AUROCs were 0.979 internally and 0.975 externally. The reported sensitivity and specificity are arithmetically consistent with patient-level counts in which the benign category is the positive class, a reading that agrees with the two false-negative AEH/cancer cases described internally and none externally. On this interpretation, which is ours, AEH or cancer was identified in 21 of 23 patients internally (91.3%; reported 95% CI, 78.2–100%) and in 16 of 16 externally. The external estimate therefore rests on 16 cases, and the method used to aggregate image-level outputs into patient-level classifications could not be identified in the accessible full text. Test-set prevalence (27% and 15%) reflects case selection, so predictive values cannot be transferred to routine practice, and the threshold was chosen by maximizing the F1 score rather than on clinical grounds. These results support discrimination for a defined binary task; they do not show that use of the system reduces missed diagnoses in practice.

The consequences of error are asymmetric. A false-negative result for AEH or cancer matters most if it leads to omission of a biopsy or delayed histologic diagnosis, whereas a false-positive result mainly increases biopsies, costs, and patient anxiety. An AI output should therefore not be used to forgo an indicated biopsy.

Broader multiclass classification has been less consistent. Zhang et al. analyzed 1851 images from 454 patients at one hospital [17]. Five-class image-level accuracy was 80.8%, compared with 90.8% when lesions were grouped as benign or premalignant/malignant (sensitivity 83.0%, specificity 96.0%). The 250 test images were drawn at random from the image pool, and patient-level separation was not reported, so the same patient may have contributed images to both training and test sets. Augmentation was applied only to the training set, poor-quality images were excluded, and the balanced test set fixed category prevalence by design. In Raimondo et al., patients rather than images were randomly allocated to training, validation, and test cohorts (1500 images from 266 consecutive patients with histologically confirmed lesions; 437 of 703 eligible patients were excluded for missing images or histology) [18]. The best configuration reported an overall image-level accuracy of 86.74%, but recall for the preneoplastic/neoplastic category was 27.59% [3,18]; that is, about seven of ten instances in the category in which missed findings matter most were not classified as such. Because patients without lesions were not included, the model was not tested across the full spectrum of diagnostic hysteroscopy. Aggregate accuracy can therefore conceal clinically important failure in a minority, high-risk class, and none of these image-based metrics should be interpreted as patient-level cancer sensitivity.

### 4.3. Micropolyps and Chronic Endometritis

Kitaya et al. developed a convolutional neural network to identify endometrial micropolyps in archived fluid-hysteroscopy images from one infertility center [19]. The final cohort comprised 208 infertile women (8443 images; 8–19 per patient), 78 with and 130 without micropolyps. The reference standard was agreement between experienced gynecologists; 31 women with intra- or inter-observer disagreement were excluded, which removes the most ambiguous cases. Test images were selected at random from the image pool, and separation of patients between training and test sets was not described. The model was evaluated on one test set and the three gynecologists on another, so the comparison was not made on the same images. Reported sensitivity, specificity, and precision were 93.6%, 92.3%, and 88.0%, respectively, and the AUROC was 0.930, which did not differ significantly from the gynecologists’ values; confidence intervals and the threshold were not reported. The reported sensitivity and specificity are arithmetically consistent with the full counts of 78 and 130 women, which leaves the unit of analysis unclear.

The clinical target needs to be stated precisely. The study classified images as showing or not showing micropolyps in women described as having histopathologically confirmed chronic endometritis, although the results report CD138-defined endometritis in 28.8% of the analyzed women; the report does not reconcile these statements. In either case, the study did not establish the sensitivity or specificity of AI for diagnosing chronic endometritis in an unselected population, and a nonsignificant difference from clinicians does not establish equivalence. Clinical adoption should not substitute this visual classification for indicated histopathologic assessment or use a negative AI result to exclude endometrial inflammation.

### 4.4. Preoperative Planning for Hysteroscopic Myomectomy

Chen et al. randomized 56 patients with submucosal myomas to standard or AI-augmented magnetic resonance imaging (MRI)-based preoperative planning [20]. Allocation used a random-number table, a single experienced surgeon performed all procedures, and blinding of the surgeon or outcome assessors was not reported. Operative time was 32.11 ± 11.86 min in the AI-assisted group and 41.32 ± 17.83 min in the control group (*p* = 0.03), a difference of about 9 min reported without a confidence interval. Median estimated blood loss was 10 mL in both groups, despite a reported distributional difference (*p* = 0.04). Agreement between preoperative and operative myoma classification (92.9% vs. 85.7%) and fluid deficit did not differ significantly. Watrowski et al. judged the trial to have some concerns with RoB 2 [3].

The small sample, single-surgeon and single-center setting, and limited outcomes restrict generalizability. Although the blood-loss distributions differed statistically, identical medians do not support a clinically meaningful reduction. The intervention used MRI segmentation for preoperative planning rather than real-time interpretation of hysteroscopic images. The trial therefore provides an early signal for a specific planning task, without establishing a broader benefit for AI-guided hysteroscopic surgery.

### 4.5. Prediction of Surgical and Reproductive Outcomes

Li et al. (2024) used a Chinese multicenter intrauterine adhesion cohort database in which 1016 patients with hysteroscopically confirmed adhesions were prospectively enrolled at two centers (Beijing, *n* = 712; Zhejiang, *n* = 304) between December 2018 and January 2020; 753 met the inclusion criteria [21]. The model combined EHR variables with second-look hysteroscopic images to predict ongoing pregnancy beyond 12 weeks within one year. Patients were randomly allocated to training (60%), validation (15%), and test (25%) sets. The authors describe the test set as external validation, but it was drawn from the same pooled database by random partitioning and therefore constitutes internal validation; it does not demonstrate transportability to independent centers. The model reported a test AUROC of 0.965 (95% CI, 0.935–0.994) and a test accuracy of 0.87, and calibration plots were presented.

Li et al. (2025) developed an image-only proportional-hazards convolutional neural network using patients from the same intrauterine adhesion database, recruited over the same period (December 2018 to January 2020), with the same inclusion criteria and ethics approvals [22]. Of 555 patients randomized 3:1 to modeling and test cohorts, 481 were analyzed after exclusions. In the test cohort, 24 of 102 patients conceived within one year; sensitivity was 95.8% (95% CI, 78.9–99.9) and specificity 98.7% (95% CI, 93.1–100), and AUROCs were 0.982, 0.992, and 0.990 in the training, validation, and test sets. Neither report states whether or to what extent patients overlap. Given the shared database, recruitment period, and inclusion criteria, the 2025 report is best regarded as a further analysis of related data rather than a new cohort, and the two reports should not be counted as independent replications. Neither study tested its model in centers outside the source database.

These studies address prognosis after treatment, not the causal effect of AI-guided treatment. Ongoing pregnancy is also distinct from live birth and perinatal outcome. The ART benefit reported in the mid-to-high-risk group (odds ratio, 6; 95% CI, 1.27–27.8) [21] derives from a stratified observational comparison with a wide confidence interval; it may reflect confounding and does not establish that model-guided referral for assisted reproduction improves live birth.

Givon et al. examined 345 consecutive hysteroscopic myomectomies in 328 women treated at one tertiary center between 2012 and 2024, using preoperative clinical and imaging variables [23]. Incomplete resection, defined as residual submucosal myoma at the end of surgery or on hysteroscopy or ultrasound within three months, occurred in 16.2% of procedures. Complete resection was coded as the positive class, and stratified five-fold patient-level cross-validation yielded an AUROC of 0.72 (95% CI, 0.65–0.79) and an average precision of 0.93. Because average precision refers to the positive class, its no-skill baseline equals the prevalence of complete resection (0.838); the value of 0.93 should not be read as precision for detecting incomplete resection. At the prespecified 0.50 threshold, sensitivity and positive predictive value for complete resection were 0.81 and 0.89, and specificity was 0.50 (95% CI, 0.36–0.63); expressed for the clinically relevant outcome, the model identified about half of the incomplete resections. Calibration was described as acceptable, but no calibration plot or statistic was reported in the Results. The model may inform counseling research, but external validation, formal calibration assessment, and evaluation of clinical utility are still required.

### 4.6. Real-Time Assistance and the Limits of Extrapolation

Detection and segmentation studies show technical feasibility for highlighting focal lesions, identifying tissue boundaries, and separating bubbles or other visual artifacts. However, processing video at an adequate frame rate does not establish safe intraoperative guidance [3]. Evaluation should include difficult views, bleeding, debris, incomplete distension, different instruments, and the effect of misleading overlays on operator decisions.

Because hysteroscopy is a digital video procedure, image overlays can be considered within the operative workflow. Routine intraoperative video capture is nonetheless not yet standard in many hysteroscopy services, and high-quality recorded video is a prerequisite for developing, validating, and auditing any real-time system; consistent recording should therefore be treated as an evaluation and governance requirement rather than an assumed input. Robotic assistance and autonomous AI represent distinct capabilities. Near-term applications are more plausibly directed toward perception, visual quality control, documentation, and support for targeted examination. Evidence from hysteroscopy cannot be generalized to the full range of minimally invasive gynecologic surgery.

## 5. From Model Performance to Clinical Translation

Discrimination, calibration, and clinical utility answer different questions. The AUROC measures ranking across possible thresholds; calibration assesses agreement between estimated and observed risks. Neither establishes that use of the model improves decisions. A diagnostic study should report clinically appropriate operating points, sensitivity and specificity with confidence intervals at those points, predictive values at the prevalence of the intended population, the consequences of false-negative and false-positive results, and performance at the intended unit of use. A prognostic model should report calibration and account for follow-up and relevant treatment exposures. Among the hysteroscopy studies in Table 4, these elements were reported incompletely and inconsistently, and statistical significance in a small trial should not be equated with clinical importance, nor a nonsignificant difference with equivalence.

Validation must prevent information from the same patient or procedure from appearing in both training and test sets. Related frames are not independent patients: frames extracted from one procedure should remain in the same partition, partitioning by patient should precede augmentation and any preprocessing fitted to the data, and a temporal or external test cohort should represent the intended setting. Leakage can inflate apparent performance by an amount that depends on the dataset and task. Curated image collections also differ from consecutive clinical encounters, in which poor-quality or uninterpretable views may be common [3]. In the studies examined here, partitioning was at the patient level in five, at the image level in two, and not applicable in the randomized planning trial (Table 3). Leakage was not demonstrated in any study, but it cannot be excluded where partitioning was by image or was not described.

Interpretability requires similar precision. Some studies provide attention maps, visualization panels, or feature-attribution analyses [22,23]. The clinical usefulness of these explanations requires evaluation. A plausible heat map does not prove that the model reasons correctly, and a feature-attribution plot does not establish calibration or freedom from confounding. Explanations should be assessed for whether they help users detect errors and make appropriate decisions.

**Figure 1:**
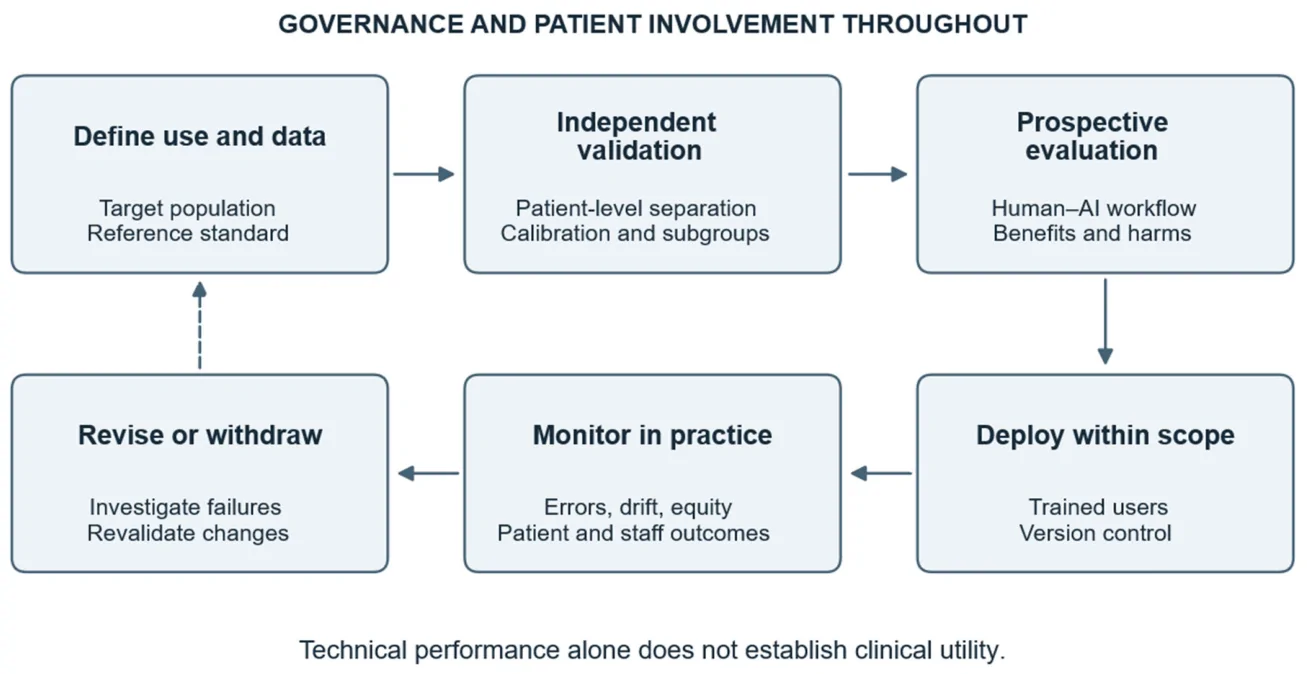
Translation pathway for clinical artificial intelligence (AI). The sequence connects a defined use case and representative data to independent validation, prospective evaluation, deployment, and monitoring. The feedback pathway requires renewed evaluation when performance or the intended use changes. Progression depends on the evidence needed for the task; governance and patient involvement extend across the entire lifecycle. Schematic created by the authors, informed by the reporting frameworks discussed in Section 5.

Reporting and appraisal tools should match the study design. The Developmental and Exploratory Clinical Investigations of DEcision support systems driven by Artificial Intelligence (DECIDE-AI) guideline addresses early clinical evaluation of AI decision support [24]. The AI extensions to the Standard Protocol Items: Recommendations for Interventional Trials (SPIRIT-AI) and the Consolidated Standards of Reporting Trials (CONSORT-AI) address trial protocols and trial reports, respectively [25,26]. The Transparent Reporting of a Multivariable Prediction Model for Individual Prognosis or Diagnosis extension (TRIPOD + AI) is the updated reporting framework for clinical prediction models using regression or machine learning [27]. QUADAS-2, PROBAST, and RoB 2 are the appraisal tools used for the relevant study types in the hysteroscopy review [28,29,30]. Reporting completeness does not by itself establish low risk of bias or readiness for care. Figure 1 illustrates a translation pathway that connects technical evaluation to prospective use and monitoring.

## 6. Governance, Safety, and Accountability

### 6.1. Governance Before Deployment

Governance should begin before procurement or patient-facing piloting. The institution should define the intended use, authorized users, data flows, clinical owner, and incident response process. A scoping review of professional acceptance identifies infrastructure, EHR integration, training, financial constraints, and uncertainty about liability and privacy as recurring implementation barriers [31]. These are operational requirements, not issues that can be resolved simply by asking clinicians to be cautious.

For systems handling patient information, institutions should review access controls, encryption, retention, subcontractors, cross-border transfers, and whether data are reused for model training. Patients should receive a clear explanation when their encounter is recorded or when AI materially affects communication. Training should use concrete examples of fabricated facts, omissions, medication errors, and inappropriate reliance on apparently confident output. Clinician sign-off should involve substantive verification rather than routine acknowledgment. Responsibilities are shared across developers, institutions, and professionals; clinical oversight does not remove vendor or institutional duties. Institutions should define, in advance and in writing, how accountability is allocated when an AI-assisted process contributes to an adverse event, covering the manufacturer’s obligations for the tool as placed on the market, the institution’s obligations for validation, integration, and monitoring, and the clinician’s responsibility for the individual clinical decision. Contracts, indemnification, insurance coverage, and incident-reporting duties should be settled before deployment rather than after an adverse event. These arrangements are governance requirements, not legal advice; applicable law and local counsel determine the final allocation of liability. These principles align with the World Health Organization’s guidance on AI for health and on large multimodal models [32,33].

### 6.2. Subgroup Performance and Equity

Performance that is acceptable on average can conceal clinically important differences between groups. Evidence from outside gynecology illustrates the mechanisms. Across five commercial speech-recognition systems, the average word error rate was 0.35 for Black speakers and 0.19 for white speakers in interviews matched for age and gender [34]. Chest radiograph classifiers underdiagnosed disease more often in female patients, Black patients, patients with low socioeconomic status, and intersectional subgroups [35], and dermatology models performed worse on images of darker skin tones [36]. These studies did not evaluate the products discussed in this review, but they show that subgroup performance must be measured rather than assumed.

The relevant subgroups depend on the modality and task. For dictation and ambient documentation, language, accent, dialect, interpreter-mediated encounters, speech impairment, and acoustic conditions are directly relevant; for patient communication, language, reading level, age, and digital access matter. For hysteroscopic image analysis, skin tone is not a direct input, whereas age and menopausal status, lesion spectrum, imaging equipment, distension medium, image quality, and operator experience are plausible sources of variation. Sex is relevant to documentation and OR tools but not to gynecologic image models. Socioeconomic status and resource setting can affect data completeness, access, and the transferability of models developed in high-resource tertiary centers. Among the hysteroscopy studies examined, one reported stratified analyses by clinical characteristics in its test cohort [22]; performance by age, equipment, or resource setting was otherwise not reported. Subgroup evaluation is therefore a requirement for future studies and local validation rather than an observed property of current systems. Dataset documentation and subgroup reporting can follow the STANDING Together recommendations [37], with acceptable performance gaps defined before deployment and monitored afterward.

### 6.3. Managing Incorrect or Incomplete AI Output

Human review is necessary but does not by itself make AI output safe: reviewers under time pressure may accept fluent but incorrect content, and omissions are harder to detect than visible errors. Services should therefore plan for incorrect or incomplete output before deployment. The following measures are proposed by the authors, consistent with the governance guidance cited above; their effectiveness in reducing AI-related harm has not been evaluated within the evidence examined here.

- **Verification of critical elements:** medications, doses, allergies, laterality, negation, and follow-up in notes; urgency and warning symptoms in patient messages; and histology, completeness of resection, fluid balance, and complications in operative reports.
- **Limits of use:** exclusion of outputs that cannot be traced to source data, tasks outside the validated scope, and patient groups not represented in local validation.
- **Escalation:** a defined route for clinicians and patients to report suspected errors and, for patient-facing tools, access to a person in urgent or uncertain situations.
- **Incident recording and surveillance:** entry of AI-related errors and near misses in the institutional reporting system, with product version and consequence, and periodic audit of outputs against source records.
- **Suspension criteria:** predefined triggers for pausing use, such as a serious system-attributable error, an error rate above a set threshold, or an unvalidated product update, with a fallback workflow.

### 6.4. Regulatory Status

Regulatory status depends on intended purpose and jurisdiction. The European Union AI Act establishes a risk-based framework; AI used in a medical device can fall within its high-risk provisions when the applicable classification conditions are met. Administrative software, documentation assistance, and diagnostic systems should not automatically be assigned the same category. Article 6 defines relevant high-risk conditions, and Article 113 sets out the phased application dates of the original regulation [38]. The AI Act was subsequently amended by Regulation (EU) 2026/1744, which, among other changes, modified the application dates of the requirements and obligations for high-risk AI systems [39]. Institutions should verify the provisions in force at deployment, including any further amendments, alongside medical-device and data-protection requirements.

In the United States, the Food and Drug Administration maintains a list of AI-enabled medical devices authorized for marketing through applicable pathways. The list is not a comprehensive inventory of all AI used in health care, and inclusion does not authorize uses beyond the particular device’s intended purpose [40]. Local assessment should distinguish regulatory authorization, technical validity, and demonstrated workflow benefit. Table 5 translates the principal risks into practical controls and monitoring measures.

## 7. Practical Implementation Roadmap

The first deployment should address a measurable need in a bounded workflow. Ambient documentation may be a suitable starting point where there is demonstrable documentation burden, adequate integration, and sufficient time for clinician review. It is not intrinsically low risk: the consequence of an error depends on what is recorded and how that record is used. Similarly, an OR scheduling recommendation may carry greater risk when it changes clinical priority than when it simply estimates room occupancy. No tool fits every setting: because performance established in one setting does not transfer automatically, validation on local and, where feasible, external representative data is a precondition for use rather than an optional refinement.

A staged implementation should proceed as follows:

1. Define the task, intended users, patient population, accountable clinical owner, and prohibited uses. Establish privacy, procurement, and regulatory requirements before piloting.
2. Measure baseline workflow performance and predefine success, suspension, and stopping criteria. Include clinically consequential errors, review burden, patient experience, and costs alongside time savings.
3. Test locally with representative data and realistic operating conditions, including the subgroups relevant to the modality. Use shadow mode where recommendations could alter diagnosis, treatment, or scheduling.
4. Pilot prospectively in a limited setting, retaining appropriate human review. Compare with usual care and document uptake, overrides, incidents, training needs, and unintended effects.
5. Decide whether to expand, revise, or stop. Expansion should require acceptable safety, useful benefit, and sustainable staffing and costs rather than favorable demonstrations alone.
6. Monitor the deployed version, subgroup performance, incidents, and drift. Revalidate material changes and maintain a practical fallback or withdrawal process.

**Figure 2:**
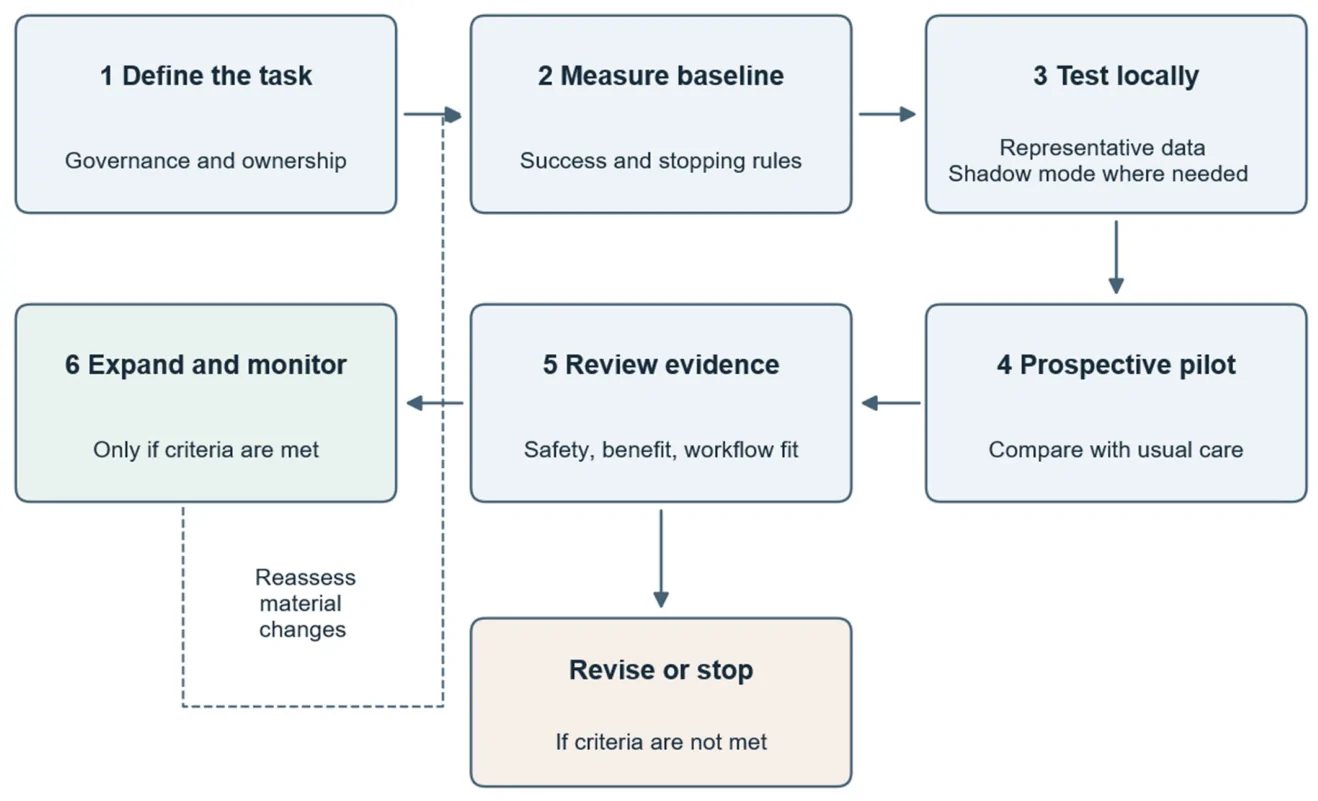
Staged implementation with an explicit decision to expand, revise, or stop. Governance precedes piloting. Expansion requires prespecified evidence of safety, benefit, and workflow fit; monitoring can trigger reassessment or withdrawal. The pathway is not a mandatory sequence of AI product types. Roadmap proposed by the authors.

These stages are shown in Figure 2. Table 6 classifies the applications discussed here by the evidence available for each task and the evaluation required before progression. Two distinctions are explicit. Ambient documentation has randomized and multisite evidence from implemented clinical settings, whereas patient-message drafting has, within the evidence examined here, only evaluator ratings of responses to public-forum questions. Drafting assistance is therefore placed in a separate category, suitable only for a tightly bounded, supervised feasibility study; routine use within a patient portal would require prospective evaluation in that setting. More generally, the categories describe a task in a defined context, not a product class. They are qualitative judgments by the authors and do not certify any commercial system; a new product version, patient population, language, or integration can change the assessment.

## 8. Discussion and Future Directions

Clinical readiness varies markedly across the applications reviewed. Documentation tools have randomized and multisite evidence from routine practice, although the size and distribution of their benefits differ by product and setting. Patient-message drafting has been evaluated, within the evidence examined here, only outside clinical workflows. Hysteroscopic image models perform well on some defined tasks, but most estimates are image-level or internally validated, patient-level error rates in high-risk categories are seldom reported, and effects on missed pathology, treatment completeness, and reproductive outcomes remain uncertain. OR models raise a different set of questions because predictions interact with scheduling policy, emergencies, staffing, and downstream capacity, and reported gains come largely from simulation.

Future documentation trials should measure serious omissions and incorrect clinical statements, not merely note completion or satisfaction. They should assess the total time needed to review and correct output and whether apparent productivity gains improve or worsen clinician workload. For patient communication, relevant outcomes include comprehension, appropriate escalation, and avoidable harm. Multilingual evaluation should address both transcription and meaning, particularly when translation is combined with summarization, and should report performance by language and accent.

Hysteroscopy research should prioritize independent multicenter validation, consecutive patient recruitment, realistic imaging conditions, patient-level partitioning and endpoints, and reporting of sensitivity, specificity, and predictive values with confidence intervals at clinically justified thresholds. Diagnostic studies need sufficient representation of premalignant and malignant disease. Prognostic studies should examine live birth and perinatal outcomes in addition to conception, report calibration in independent cohorts, and state explicitly whether cohorts overlap with earlier reports; randomized impact studies are needed before model predictions are used to select treatment. OR evaluations should prospectively test clinical access, cancellations, overtime, response to emergencies and overruns, staff experience, and downstream displacement of workload. Economic analyses should include licensing, integration, training, review time, and ongoing monitoring.

This review has limitations. Its targeted selection can introduce selection and publication bias, it is not an exhaustive systematic assessment, and no screening log or record counts are available for the original searches. Some values were taken from the secondary review rather than from the primary report. Formal risk-of-bias judgments were taken from an existing specialty review rather than independently repeated, and our descriptive appraisal is not a substitute for duplicate formal assessment. Studies vary in design, product version, outcome definition, and validation strategy, precluding meaningful pooled estimates or direct ranking across applications. Evidence may overlap across reviews and research cohorts. Finally, technical development and regulatory requirements evolve rapidly; the framework should guide reassessment rather than fix a permanent hierarchy of technologies.

## 9. Conclusions

For selected services, clinician-reviewed dictation and ambient documentation are practical starting points supported by randomized trials and multisite observational data from routine practice. Their benefits remain product- and setting-dependent, and inaccuracies and review burden still matter. Patient-message drafting warrants only bounded, supervised feasibility testing until it has been evaluated in implemented portals; summarization and reporting tools require source verification and accountable professional review. Hysteroscopic models show promising discrimination for specific tasks, but current evidence, which is largely retrospective, image-level, or internally validated, does not support autonomous diagnosis, replacement of indicated histopathology, or treatment decisions based on unvalidated predictions. Predictive and optimization tools may improve OR organization if they are built on reliable local data, accommodate emergencies and disruptions, and are tested prospectively within the actual workflow. Adoption should begin with a defined need and baseline measurement, proceed through subgroup-aware local validation and monitored piloting, and include clear criteria for expansion, suspension, or withdrawal.

## Figures and Tables

**Table 1:** AI applications across the clinical workflow and suggested local evaluation outcomes.

Application	Potential Value	Main Limitation	Local Outcome to Measure
Dictation and ambient documentation	Less manual note creation	Transcription, omission, and fabrication errors	Total documentation and review time; consequential errors
Chart summarization	Faster access to longitudinal information	Loss of clinically relevant context	Source fidelity; omitted high-risk information
Patient-message drafting	Support for clear, timely communication	Incorrect advice or urgency assessment	Clinician editing; comprehension; appropriate escalation
Operative reporting and registries	Structured capture; less duplicate entry	Propagation of unsupported data	Field-level accuracy; missing data; audit discrepancies
Preoperative planning	Support for localization and planning	Limited comparative clinical evidence	Procedure-specific outcomes and planning errors
Hysteroscopic image analysis	Lesion recognition and visual support	Limited independent prospective validation	Patient-level false negatives; biopsy and treatment outcomes
OR prediction and scheduling	Better estimates and resource allocation	Transferability; emergencies and downstream constraints	Cancellations, delays, overtime, access, and staff workload

Synthesis by the authors of the applications discussed in Section 3 and Section 4. Potential value should not be interpreted as established effectiveness. AI, artificial intelligence; OR, operating room.

**Table 2:** Selected quantitative evidence outside hysteroscopy, with the setting in which each result was obtained.

Study and Design	Evidence Setting	Population or Dataset	Selected Result	Interpretive Limit
Rotenstein et al. [4]; multisite longitudinal cohort	Routine clinical practice; observational	8581 clinicians; 1809 adopters; 5 institutions	13.4 min less EHR time and 16.0 min less documentation time per 8 scheduled patient hours; +0.49 visits/week	Associations; voluntary adoption; no significant after-hours reduction
Afshar et al. [5]; stepped-wedge randomized trial	Routine clinical practice; randomized	66 practitioners; 24 weeks	0.36 fewer hours/day on notes; reduced exhaustion/disengagement	After-hours finding sensitive to outliers; fulfillment endpoint not significant by trial criterion
Lukac et al. [6]; three-group randomized trial	Routine clinical practice; randomized	238 physicians; 14 specialties	Time-in-note: Nabla −9.5%; DAX −1.7% versus control	Only Nabla effect significant; occasional inaccuracies
Ayers et al. [12]; cross-sectional evaluation	Outside clinical workflow; public-forum questions	195 questions; 585 evaluations	Chatbot preferred in 78.6% of evaluations	Professional raters; no patient portal; no clinical outcomes
Çalışkan et al. [15]; discrete-event simulation	Simulation	500 replications; stylized general-surgery OR	Delay −42.9%; overtime −46.0%; cancellations −54.8%	Simulated relative gains; no clinical deployment

Values retain the outcome definitions of their source studies and should not be compared as common effect sizes. DAX, Dragon Ambient eXperience; EHR, electronic health record; OR, operating room.

**Table 3:** Primary hysteroscopy studies: task, design, data, and validation.

Study	Task And Intended Use	Design, Setting, Period	Patients; Images or Procedures	Unit of Analysis	Reference Standard; Comparator	Validation; Data Separation
Wang et al. [16]	Binary AEH/EC vs. benign; diagnostic support	Retrospective; 3 tertiary hospitals, China; training 2008–2017; tests 2018–2019	1394 patients; 55,874 images (training 1204; internal test 85; external test 105)	Image input; test metrics consistent with patient counts (aggregation method NV)	Category confirmed by two experts (histologic basis NV); 12 endoscopists	Temporal internal and geographic external tests; no case overlap reported
Zhang et al. [17]	Five-class and binary lesion classification	Retrospective; single center, China; 2017–2019	454 patients; 1851 images; test 250 images	Image	Histopathology; 3 gynecologists	Random image-level split; patient separation not reported; augmentation of training set only
Raimondo et al. [18]	Lesion identification and 3-class classification	Retrospective, consecutive; single center, Italy; January–May 2021	266 of 703 patients; 1500 images	Image (patient-level allocation)	Histopathology; none	Random patient-level split (157/54/55); internal only
Kitaya et al. [19]	Detection of micropolyps in infertile women with chronic endometritis	Retrospective in silico; single infertility center, Japan; April 2019–December 2021; registered (UMIN000047385)	208 women (78 with micropolyps); 8443 images (8–19 per patient)	Image selection for test sets; reported metrics consistent with patient counts (unit unclear)	Agreement of experienced gynecologists (31 women with intra- or inter-observer disagreement excluded); 3 gynecologists, assessed on a different test set	Random image-level selection of test images; patient separation not described; augmentation for training
Chen et al. [20]	MRI segmentation for preoperative planning of myomectomy	Randomized (random-number table); single center, China; 2022–2023; one surgeon	56 patients (28 vs. 28)	Patient	Operative FIGO type; standard MRI planning	Not applicable (impact study); segmentation model previously published
Li et al., 2024 [21]	One-year conception after adhesiolysis (EHR + images)	Prospective database; 2 centers, China; December 2018–January 2020	753 patients; 5014 images	Patient	Ongoing pregnancy >12 weeks; AFS, CSGE, other models	Random patient-level split 60/15/25 of pooled data; internal (described as external by authors)
Li et al., 2025 [22]	One-year conception after adhesiolysis (images only)	Same database and recruitment period as [21]	555 randomized, 481 analyzed; 4922 images	Patient	Ongoing pregnancy ≥12 weeks; InceptionV3, AFS, CSGE, endometrial thickness	Random patient-level split 3:1; internal; overlap with [21] likely, not quantified
Givon et al. [23]	Preoperative prediction of incomplete myomectomy	Retrospective, consecutive; single tertiary center, Israel; 2012–2024	328 women; 345 procedures	Procedure	Residual myoma at end of surgery or on hysteroscopy or ultrasound within 3 months; logistic regression	Stratified 5-fold patient-level cross-validation; no external test

AEH, atypical endometrial hyperplasia; AFS, American Fertility Society score; CSGE, Chinese Society of Gynecological Endoscopy score; EC, endometrial cancer; EHR, electronic health record; FIGO, International Federation of Gynecology and Obstetrics; MRI, magnetic resonance imaging; NV, not verifiable in the sources accessible to the authors (full text not accessible or item not located).

**Table 4:** Primary hysteroscopy studies: reported metrics, clinical outcomes, limitations, and source of information.

Study	Reported Metrics (95% CI; Threshold; Prevalence)	Clinical Outcomes Assessed	Principal Limitation	Source of Information
Wang et al. [16]	AUROC 0.979 (0.942–1.000) internal, 0.975 (0.942–0.998) external. AEH/EC classified correctly in 21/23 (internal) and 16/16 (external) patients (authors’ reading of reported values). Threshold: maximum F1 on a validation set. AEH/EC prevalence 27% and 15%. Predictive values reported in tables, NV.	None	Retrospective; 16 external AEH/EC cases; patient selection and aggregation method NV	Full text
Zhang et al. [17]	Five-class accuracy 80.8%; binary sensitivity 83.0%, specificity 96.0%, AUROC 0.944 (image level; CIs not reported). Test prevalence fixed by design (40% premalignant/malignant images).	None	Possible patient overlap; curated images; single device	Full text
Raimondo et al. [18]	Overall accuracy 86.74% (with clinical factors); recall for preneoplastic/neoplastic class 27.59% (image level; CIs not reported). Prevalence of (pre)neoplastic lesions 20.7% of patients.	None	Lesion-only cohort; 62% of eligible patients excluded; low recall in the high-risk class	Full text
Kitaya et al. [19]	Sensitivity 93.6%, specificity 92.3%, precision 88.0%, AUROC 0.930; CIs and threshold not reported. Micropolyp prevalence 37.5% of women; CD138-defined chronic endometritis reported in 28.8%.	None	Easy-case selection (disagreements excluded); CNN and clinicians tested on different image sets; target is a visual sign, not a diagnosis of endometritis	Full text
Chen et al. [20]	Operative time 32.11 ± 11.86 vs. 41.32 ± 17.83 min (*p* = 0.03; CI not reported); blood loss median 10 mL in both groups (*p* = 0.04); type agreement 92.9% vs. 85.7% (not significant); fluid deficit not significant.	Operative time, blood loss, fluid deficit, complications	Small; single surgeon; blinding and registration not reported; RoB 2 some concerns [3]	Full text
Li et al., 2024 [21]	Test AUROC 0.965 (0.935–0.994); test accuracy 0.87; C-index 0.93 (0.90–0.95); calibration plots and decision curves presented. ART benefit in mid-to-high-risk group: odds ratio 6 (1.27–27.8).	Ongoing pregnancy; not live birth	Internal validation; overlap with [22]; stratified ART comparison is observational	Full text
Li et al., 2025 [22]	Test AUROC 0.990 (0.974–1.000); sensitivity 95.8% (78.9–99.9) and specificity 98.7% (93.1–100) for one-year conception; positive and negative predictive values 95.8% and 98.7%; 24/102 test patients conceived within one year.	Ongoing pregnancy; not live birth	Same source as [21]; exclusions after randomization; few events	Full text
Givon et al. [23]	AUROC 0.72 (0.65–0.79); average precision 0.93 with complete resection as positive class (prevalence baseline 0.838). At threshold 0.50: sensitivity 0.81 (0.77–0.86), positive predictive value 0.89 (0.85–0.92), and specificity 0.50 (0.36–0.63) for complete resection; that is, about half of incomplete resections were identified. Incomplete resection 16.2%. Calibration described as acceptable, without a plot or statistic in the Results.	Resection completeness	Single center; no external validation; positive-class coding; missing imaging data	Full text

Metrics are reported as defined in each source and are not comparable across tasks. The no-skill baseline for average precision equals the prevalence of the positive class. ART, assisted reproductive technology; AUROC, area under the receiver operating characteristic curve; C-index, concordance index; CI, confidence interval; NV, not verifiable in the sources accessible to the authors; RoB 2, revised Cochrane risk-of-bias tool for randomized trials; CNN, convolutional neural network.

**Table 5:** Practical risk controls and monitoring measures for local implementation.

Risk	Control Before Use	Monitoring After Deployment
Fabricated or omitted content	Source verification of predefined critical elements before release	Consequential error and correction rates; audit against source records
Speech or translation errors	Testing across the languages, accents, and acoustic conditions of the service	Medication, dose, negation, and attribution errors
Incorrect output reaching the record or the patient	Limits of use; escalation routes; fallback workflow; predefined suspension criteria	Incident and near-miss reports; time to correction; suspension events
Privacy breach or unauthorized reuse	Approved data flows, retention rules, access controls, and vendor terms	Access logs; retention compliance; security incidents
Automation bias	Training with error examples; explicit escalation and override routes	Appropriate overrides; missed errors; user behavior
Unequal performance	Representative validation; subgroup assessment relevant to the modality; predefined acceptable gaps	Subgroup error rates, access, and patient experience
Poor workflow integration	Usability testing; prospective pilot; fallback process	Review time; duplicate entry; adoption and abandonment
Model or software change	Version control; predefined change and revalidation policy	Drift; vendor updates; unexpected output changes
Unclear accountability	Named institutional and clinical owners; incident process	Incident response, corrective actions, and unresolved issues

Controls proposed by the authors, informed by the evidence and guidance discussed in Section 5 and Section 6. Human review is one safeguard within a wider governance system.

**Table 6:** Task-specific readiness framework proposed by the authors.

Category	Examples in This Review	Evidence Examined in This Review	Condition for Use or Progression
Supervised local pilot supported by implementation evidence	Clinician-reviewed dictation or ambient notes	Randomized trials and a multisite observational study in routine practice [4,5,6]	Approved product and data handling; trained reviewers; baseline measurement; predefined benefit, safety, and stopping criteria
Bounded, supervised feasibility study only	Clinician-edited drafts of patient messages, none released without review	Evaluator ratings of responses to public-forum questions [12]; no evidence from an implemented portal	Limited scope and volume; review and editing of every draft; error and escalation monitoring; prospective evaluation before any routine portal use
Requires substantial institutional integration and prospective local evaluation	Chart summaries; routine portal deployment; OR duration or cancellation prediction	Commentary and proposed uses [11]; systematic reviews of predictive models [13,14]	Reliable source data; workflow integration; local validation against current practice; prospective evaluation
Investigational clinical application	Hysteroscopic diagnostic support; AI-assisted MRI planning; fertility prediction; unvalidated operative-data extraction	Retrospective or internally validated studies and one small randomized planning trial (Table 3 and Table 4)	Independent validation; patient-level metrics; appropriate oversight; prospective assessment of clinical utility
Not supported for routine autonomous use	Unsupervised image diagnosis; real-time autonomous operative guidance; autonomous high-impact OR scheduling	No direct clinical evidence; simulation only for scheduling [15]	Research evaluation and applicable authorization before clinical use; no extrapolation from simulations

These categories are qualitative implementation judgments, not formal evidence grades or regulatory classifications. Product version, intended purpose, population, and consequences of error determine the final local assessment. AI, artificial intelligence; MRI, magnetic resonance imaging; OR, operating room.

## Data Availability

No new data were created or analyzed in this study. Data sharing is not applicable to this article.

## Abbreviations

AEH, atypical endometrial hyperplasia; AFS, American Fertility Society; AI, artificial intelligence; ART, assisted reproductive technology; AUROC, area under the receiver operating characteristic curve; CI, confidence interval; CONSORT-AI, Consolidated Standards of Reporting Trials–Artificial Intelligence extension; CSGE, Chinese Society of Gynecological Endoscopy; DAX, Dragon Ambient eXperience; DECIDE-AI, Developmental and Exploratory Clinical Investigations of DEcision support systems driven by Artificial Intelligence; EC, endometrial cancer; EHR, electronic health record; FIGO, International Federation of Gynecology and Obstetrics; LLM, large language model; MRI, magnetic resonance imaging; NV, not verifiable in the accessible sources; OR, operating room; PROBAST, Prediction Model Risk of Bias Assessment Tool; QUADAS-2, Quality Assessment of Diagnostic Accuracy Studies 2; RoB 2, revised Cochrane risk-of-bias tool for randomized trials; SPIRIT-AI, Standard Protocol Items: Recommendations for Interventional Trials–Artificial Intelligence extension; TRIPOD + AI, Transparent Reporting of a Multivariable Prediction Model for Individual Prognosis or Diagnosis–Artificial Intelligence update.

## References

[1] Sahni N.R., Carrus B. (2023). Artificial Intelligence in U.S. Health Care Delivery. N. Engl. J. Med..

[2] Angus D.C., Khera R., Lieu T., Liu V., Ahmad F.S., Anderson B., Bhavani S.V., Bindman A., Brennan T., Celi L.A. (2025). AI, Health, and Health Care Today and Tomorrow: The JAMA Summit Report on Artificial Intelligence. JAMA.

[3] Watrowski R., Di Spiezio Sardo A., Török P., Rosati A., Kostov S., Alkatout I., Vitale S.G. (2026). Artificial Intelligence, Deep Learning, and Computer Vision in Hysteroscopy: A Systematic Review. Diagnostics.

[4] Rotenstein L.S., Holmgren A.J., Thombley R., Sriram A., Dbouk R.H., Jost M., Aizenberg D., MacDonald S., Kanaparthy N., Williams B. (2026). Changes in Clinician Time Expenditure and Visit Quantity with Adoption of Artificial Intelligence–Powered Scribes: A Multisite Study. JAMA.

[5] Afshar M., Baumann M.R., Resnik F., Hintzke J., Sullivan A.G., Wills G., Lemmon K., Dambach J., Mrotek L.A., Quinn M. (2025). A Pragmatic Randomized Controlled Trial of Ambient Artificial Intelligence to Improve Health Practitioner Well-Being. NEJM AI.

[6] Lukac P.J., Turner W., Vangala S., Chin A.T., Khalili J., Shih Y.C.T., Sarkisian C., Cheng E.M., Mafi J.N. (2025). Ambient AI Scribes in Clinical Practice: A Randomized Trial. NEJM AI.

[7] Shah S.J., Devon-Sand A., Ma S.P., Jeong Y., Crowell T., Smith M., Liang A.S., Delahaie C., Hsia C., Shanafelt T. (2025). Ambient artificial intelligence scribes: Physician burnout and perspectives on usability and documentation burden. J. Am. Med. Inform. Assoc..

[8] Ng J.J.W., Wang E., Zhou X., Zhou K.X., Goh C.X.L., Sim G.Z.N., Tan H.K., Goh S.S.N., Ng Q.X. (2025). Evaluating the performance of artificial intelligence-based speech recognition for clinical documentation: A systematic review. BMC Med. Inform. Decis. Mak..

[9] Alboksmaty A., Aldakhil R., Hayhoe B.W.J., Ashrafian H., Darzi A., Neves A.L. (2025). The impact of using AI-powered voice-to-text technology for clinical documentation on quality of care in primary care and outpatient settings: A systematic review. EBioMedicine.

[10] Atiku S., Owolanke K., Olakotan O. (2026). Assessing the Reliability, Accuracy, and Relevance of Artificial Intelligence Speech Recognition for Clinical Documentation: A Scoping Review. J. Eval. Clin. Pract..

[11] Maw A., Pandita A., Burden M. (2026). Hospitalists Are Already Using AI—Why Implementation Will Determine Its Impact. J. Med. Internet Res..

[12] Ayers J.W., Poliak A., Dredze M., Leas E.C., Zhu Z., Kelley J.B., Faix D.J., Goodman A.M., Longhurst C.A., Hogarth M. (2023). Comparing Physician and Artificial Intelligence Chatbot Responses to Patient Questions Posted to a Public Social Media Forum. JAMA Intern. Med..

[13] Bellini V., Russo M., Domenichetti T., Panizzi M., Allai S., Bignami E.G. (2024). Artificial Intelligence in Operating Room Management. J. Med. Syst..

[14] Bellini V., Guzzon M., Bigliardi B., Mordonini M., Filippelli S., Bignami E. (2020). Artificial Intelligence: A New Tool in Operating Room Management. Role of Machine Learning Models in Operating Room Optimization. J. Med. Syst..

[15] Çalışkan Y.K., Başak F., Erdem O., Kudaş İ. (2026). Beyond block time: A head-to-head comparison of reinforcement learning, genetic algorithms, and predict-then-optimize scheduling for operating room workflow using discrete-event simulation. Int. J. Med. Inform..

[16] Wang W., Cai Y., Guo Z., Zhao A., Ma W., Wang W., Wang S., Zhu X., Du X., Shen W. (2025). A deep learning-based computer-aided diagnosis system for detecting atypical endometrial hyperplasia and endometrial cancer through hysteroscopy. iScience.

[17] Zhang Y., Wang Z., Zhang J., Wang C., Wang Y., Chen H., Shan L., Huo J., Gu J., Ma X. (2021). Deep learning model for classifying endometrial lesions. J. Transl. Med..

[18] Raimondo D., Raffone A., Salucci P., Raimondo I., Capobianco G., Galatolo F.A., Cimino M.G.C.A., Travaglino A., Maletta M., Ferla S. (2024). Detection and Classification of Hysteroscopic Images Using Deep Learning. Cancers.

[19] Kitaya K., Yasuo T., Yamaguchi T., Morita Y., Hamazaki A., Murayama S., Mihara T., Mihara M. (2024). Construction of deep learning-based convolutional neural network model for automatic detection of fluid hysteroscopic endometrial micropolyps in infertile women with chronic endometritis. Eur. J. Obstet. Gynecol. Reprod. Biol..

[20] Chen M., Kong W., Li B., Tian Z., Yin C., Zhang M., Pan H., Bai W. (2023). Revolutionizing hysteroscopy outcomes: AI-powered uterine myoma diagnosis algorithm shortens operation time and reduces blood loss. Front. Oncol..

[21] Li B., Chen H., Lin X., Duan H. (2024). Multimodal learning system integrating electronic medical records and hysteroscopic images for reproductive outcome prediction and risk stratification of endometrial injury: A multicenter diagnostic study. Int. J. Surg..

[22] Li B., Chen H., Duan H. (2025). Visualized hysteroscopic artificial intelligence fertility assessment system for endometrial injury: An image-deep-learning study. Ann. Med..

[23] Givon I., Sabag D.N., Yacobi B., Chaim O.B., Bor N., Matot R., Nassie D., Borovich A. (2026). Machine Learning Prediction of Incomplete Hysteroscopic Myomectomy Using Preoperative Clinical and Imaging Variables. J. Minim. Invasive Gynecol..

[24] Vasey B., Nagendran M., Campbell B., Clifton D.A., Collins G.S., Denaxas S., Denniston A.K., Faes L., Geerts B., Ibrahim M. (2022). Reporting guideline for the early stage clinical evaluation of decision support systems driven by artificial intelligence: DECIDE-AI. BMJ.

[25] Cruz Rivera S., Liu X., Chan A.-W., Denniston A.K., Calvert M.J., SPIRIT-AI and CONSORT-AI Working Group (2020). Guidelines for clinical trial protocols for interventions involving artificial intelligence: The SPIRIT-AI extension. Nat. Med..

[26] Liu X., Cruz Rivera S., Moher D., Calvert M.J., Denniston A.K., SPIRIT-AI and CONSORT-AI Working Group (2020). Reporting guidelines for clinical trial reports for interventions involving artificial intelligence: The CONSORT-AI extension. Nat. Med..

[27] Collins G.S., Moons K.G.M., Dhiman P., Riley R.D., Beam A.L., Van Calster B., Ghassemi M., Liu X., Reitsma J.B., van Smeden M. (2024). TRIPOD+AI statement: Updated guidance for reporting clinical prediction models that use regression or machine learning methods. BMJ.

[28] Whiting P.F., Rutjes A.W.S., Westwood M.E., Mallett S., Deeks J.J., Reitsma J.B., Leeflang M.M.G., Sterne J.A.C., Bossuyt P.M.M., QUADAS-2 Group (2011). QUADAS-2: A revised tool for the quality assessment of diagnostic accuracy studies. Ann. Intern. Med..

[29] Wolff R.F., Moons K.G.M., Riley R.D., Whiting P.F., Westwood M., Collins G.S., Reitsma J.B., Kleijnen J., Mallett S., PROBAST Group (2019). PROBAST: A Tool to Assess the Risk of Bias and Applicability of Prediction Model Studies. Ann. Intern. Med..

[30] Sterne J.A.C., Savović J., Page M.J., Elbers R.G., Blencowe N.S., Boutron I., Cates C.J., Cheng H.Y., Corbett M.S., Eldridge S.M. (2019). RoB 2: A revised tool for assessing risk of bias in randomised trials. BMJ.

[31] Nesa L., Rony M.K.K., Chowdhury S., Naznin M.B., Halder K., Ara M.H., Akter N.N., Mankhin K., Shabnur J.M., Alam J. (2025). Artificial Intelligence in Healthcare: A Scoping Review of Medical Professionals’ Acceptance and Institutional Challenges in Implementation. J. Eval. Clin. Pract..

[32] World Health Organization (2021). Ethics and Governance of Artificial Intelligence for Health: WHO Guidance.

[33] World Health Organization (2024). Ethics and Governance of Artificial Intelligence for Health: Guidance on Large Multi-Modal Models.

[34] Koenecke A., Nam A., Lake E., Nudell J., Quartey M., Mengesha Z., Toups C., Rickford J.R., Jurafsky D., Goel S. (2020). Racial disparities in automated speech recognition. Proc. Natl. Acad. Sci. USA.

[35] Seyyed-Kalantari L., Zhang H., McDermott M.B.A., Chen I.Y., Ghassemi M. (2021). Underdiagnosis bias of artificial intelligence algorithms applied to chest radiographs in under-served patient populations. Nat. Med..

[36] Daneshjou R., Vodrahalli K., Novoa R.A., Jenkins M., Liang W., Rotemberg V., Ko J., Swetter S.M., Bailey E.E., Gevaert O. (2022). Disparities in dermatology AI performance on a diverse, curated clinical image set. Sci. Adv..

[37] Alderman J.E., Palmer J., Laws E., McCradden M.D., Ordish J., Ghassemi M., Pfohl S.R., Rostamzadeh N., Cole-Lewis H., Glocker B. (2025). Tackling algorithmic bias and promoting transparency in health datasets: The STANDING Together consensus recommendations. Lancet Digit. Health.

[38] European Parliament, Council of the European Union (2024). Regulation (EU) 2024/1689 of 13 June 2024 Laying Down Harmonised Rules on Artificial Intelligence (Artificial Intelligence Act). Off. J. Eur. Union.

[39] European Parliament, Council of the European Union (2026). Regulation (EU) 2026/1744 of 8 July 2026 Amending Regulations (EU) 2024/1689, (EU) 2018/1139 and (EU) 2023/1230 as Regards the Simplification of the Implementation of Harmonised Rules on Artificial Intelligence (Digital Omnibus on AI). Off. J. Eur. Union.

[40] U.S. Food and Drug Administration Artificial Intelligence-Enabled Medical Devices. https://www.fda.gov/medical-devices/software-medical-device-samd/artificial-intelligence-enabled-medical-devices.

